# The Genetic Changes of Hepatoblastoma

**DOI:** 10.3389/fonc.2021.690641

**Published:** 2021-07-21

**Authors:** Huitong Chen, Qian Guan, Huiqin Guo, Lei Miao, Zhenjian Zhuo

**Affiliations:** ^1^ Department of Pediatric Surgery, Guangzhou Institute of Pediatrics, Guangdong Provincial Key Laboratory of Research in Structural Birth Defect Disease, Guangzhou Women and Children’s Medical Center, Guangzhou Medical University, Guangzhou, China; ^2^ School of Medicine, South China University of Technology, Guangzhou, China

**Keywords:** hepatoblastoma, etiology, genetics, single nucleotide polymorphism, epidemiology

## Abstract

Hepatoblastoma is the most common malignant liver cancer in childhood. The etiology of hepatoblastoma remains obscure. Hepatoblastoma is closely related to genetic syndromes, hinting that hepatoblastoma is a genetic predisposition disease. However, no precise exposures or genetic events are reported to hepatoblastoma occurrence. During the past decade, significant advances have been made in the understanding of etiology leading to hepatoblastoma, and several important genetic events that appear to be important for the development and progression of this tumor have been identified. Advances in our understanding of the genetic changes that underlie hepatoblastoma may translate into better patient outcomes. Single nucleotide polymorphisms (SNPs) have been generally applied in the research of etiology’s exploration, disease treatment, and prognosis assessment. Here, we reviewed and discussed the molecular epidemiology, especially SNPs progresses in hepatoblastoma, to provide references for future studies and promote the study of hepatoblastoma’s etiology.

## Introduction

Hepatoblastoma arising from the hepatocyte precursor is the most common malignant liver tumor among children (1). The typical clinical symptoms of hepatoblastoma are alpha-fetoprotein (AFP) rising and abdominal mass (2). Due to the rarity of hepatoblastoma, diagnosis and treatment are facing challenges. With medical-technical development such as adjuvant chemotherapy and hepatectomy in decades, the 5-year survival rate is greater than 70% nowadays (3, 4). Despite the improved survival rate, numerous survivors suffer treatment-related side effects, such as hearing loss or cardiomyopathy (5). In addition, the prognosis of advanced stage hepatoblastoma patients with unresectable tumors remains poor (6).

Unlike the hepatocellular carcinoma which has clear pathogenesis (HCC), the etiology of hepatoblastoma has no connection with hepatitis B virus or cirrhosis (7). The first study of genetic-molecular changes in hepatoblastoma was conducted in the late 1980s (8). However, the etiology of hepatoblastoma remains unclear by far. In this review, we aimed at giving a brief overview of the molecular epidemiology for hepatoblastoma, focusing on the SNPs that influence hepatoblastoma risk. We further discussed the clinical challenges for elucidating the etiology of hepatoblastoma and provided theoretical basis for future prevention, diagnosis, and therapeutic approaches for hepatoblastoma.

## Epidemiology

Because of the rarity of hepatoblastoma, the hepatoblastoma’s epidemiology has not been investigated comprehensively. Majority of hepatoblastoma are sporadic and are commonly found in children in their first 5 years, with a predominance in boys (9). The incidence of hepatoblastoma remains at a lower level worldwide comparing with other solid tumors in children, including neuroblastoma and Wilms tumor (10–12). Employing the Surveillance, Epidemiology and End Results (SEER) database, the incidence of hepatoblastoma in the United States was 1.5–1.9 per million with an upward tendency (5, 13). It is similar to the incidence rate of roughly 1.1 per million in China and 1.7 per million in the Nordic countries (14, 15). There is little difference of hepatoblastoma’s incidence in diverse countries, but these data were not collected from the same period. The difference in ethnicity is also correlated to the incidence rate. It was reported that the incidence rate in blacks was relative lower (16). A population-based analysis conducted in the United States revealed that the higher the maternal education, the lower the incidence rate is of hepatoblastoma (17).

## Genetic Syndromes

Although most hepatoblastomas are sporadic (18), hepatoblastoma was reported to be closely related to genetic syndromes, including familial adenomatous polyposis (FAP), Beckwith-Wiedemann syndrome (BWS), and trisomy 18 (19–21).

The association between FAP and hepatoblastoma was originally perceived in 1983 (22). Mutation of the *APC* was detected in hepatoblastoma patients with a family history of FAP (23). *APC* mutation was also found in other cancers, including gastric and colorectal cancer (24, 25). However, in subsequent research, Harvey et al. demonstrated that no *APC* mutation was found in sporadic hepatoblastoma (26). BWS, an overgrowth syndrome associated with alteration of genomic imprinting on chromosome 11p15.5, is characterized by macroglossia, high birth weight, overgrowth of abdominal organs, and neonatal hypoglycemia (27, 28). Comparing with children without BWS, the relative rate of hepatoblastoma among children with BWS was 2,280 (95% CI: 928–11,656) (29). Trisomy 18, regarded as a fatal disease, affects approximately 1 per 6,000 newborns (30). The correlation between trisomy 18 and hepatoblastoma has been reported in several previous studies (21, 31–33). Tomlinson et al. raised an interesting point that the hepatoblastoma cases with trisomy 18 almost were females, and this situation was contrary to hepatoblastoma with higher prevalence in males (34). In addition to the syndromes mentioned above, some genetic syndromes have been reported, including Prader-Willi syndrome, and Simpson-Golabi-Behmel syndrome (35–37) (**Table 1**). However, these cases are too rare to consider as a close risk factor for hepatoblastoma.

**Table 1:** Genetic syndromes of hepatoblastoma.

Syndrome	Chromosome	Correlated gene	Relative risk	Reference
**FAP**	5q21	*APC*	750–7,500	(38)
**BWS**	11p15.5	IGF2-H19	2280	(29)
**Trisomy 18**	18	/	Unknown	(21)
**SGBS**	Xq26	*GPC3*	Unknown	(37)
**PWS**	46XY del (15) (q11, q13)	/	Unknown	(36)

FAP, familial adenomatous polyposis; BWS, Beckwith-Wiedemann syndrome; SGBS, Simpson-Golabi-Behmel syndrome; PWS, Prader-Willi syndrome.

## Risk Factors

Although the etiology of hepatoblastoma remains unclear, some risk factors have been identified (**Table 2**). The United Kingdom Childhood Cancer Study (UKCCS) reported that parental smoking is a risk factor for hepatoblastoma (OR = 4.74, 95% CI:1.68–13.35), although only 28 hepatoblastoma patients were recruited in this study (39). The result of subsequent studies from the United States and China also supported this conclusion (40, 41). In 2009, parental smoking has been declared as the significant high-risk factor for hepatoblastoma by the International Agency for Research on Cancer (47).

**Table 2:** Risk factors of hepatoblastoma.

Risk factors	Location/category	OR	95% CI	Reference
**Parental smoking**	UK	4.74	1.68–13.35	(39)
	China	2.9	1.1–4.2	(40)
	US	2.69	1.18–6.13	(41)
**VLBW**	US	50.57	6.59–387.97	(42)
	China	26.0	14.0–65.7	(40)
	Japan	15.6	7.6–31.1	(43)
	Nordic countries	9.5	2.3–38.2	(15)
**Parental occupational exposure**	Paints	1.71	1.04–2.81	(44)
	Wood dust	2.41	0.99–5.88	(45)
	Metal fumes	8.0	1.5–148.4	(46)
	Petroleum	2.3	1.2–4.6	(46)

As mentioned above, overgrowth syndrome is firmly correlated with hepatoblastoma; not only that, premature birth and very low birth weight (VLBW, <1,500 g) is closely associated with hepatoblastoma. The data analyzation, which was from California’s population-based cancer registry, indicated that hepatoblastoma risk is remarkably increase in VLBW children (OR=50.57, 95% CI: 6.59–387.97) (42). Although the ORs were diverse in different regions, whole ORs were greater than one and proved VLBW was an obvious risk factor for hepatoblastoma (15, 40, 43).

Janitz et al. had confirmed that maternal and paternal occupational exposures to paints were etiologically relevant to hepatoblastoma (44). Other studies indicated that parental occupational exposures to wood dust, metal fumes, and petroleum products also could be the risk factors (45, 46). On account of the rarity of hepatoblastoma, the investigations of risk factors are relatively limited.

## Single Nucleotide Polymorphisms With Hepatoblastoma

In the late 1990s, research showed that the existence of mutation of *CTNNB1* (β-catenin gene) might lead to β-catenin accumulation, resulting in the development of hepatoblastoma (48). Some pathways related to hepatoblastoma molecular mechanisms were detected, including the well-studied Wnt/β-catenin and MYC pathways (49). However, research on the molecular basis in hepatoblastoma was limited. It is urgent to identify early diagnostic molecular-genetic markers for timely and valid therapeutic choices.

Single nucleotide polymorphisms (SNPs), comparing with rare gene mutation, were identified to abundantly exist in human genome by Human Genome Project (50). The susceptibilities and pathogenesis of disease and genetic heterogeneity are tightly correlated with SNPs (51–54). When present in non-coding regions, SNPs are regarded as critical genetic markers and can regulate protein expression (55). SNPs have been generally applied in the research of etiology’s exploration, treatment of disease, and prognosis assessment. Comparing to other solid tumors in children, studies of the association between hepatoblastoma and gene polymorphism are relatively few. Here we summarized the significant hepatoblastoma susceptibility SNPs in **Table 3**.

**Table 3:** Summary of hepatoblastoma susceptibility SNPs.

Chromosome	Variant	Candidate gene	Alternate allele	Effect	Reference
**17q23**	G-463-A	MPO	G>A	Protective factor	(56)
**11q13**	rs9344	CCND1	G>A	Risk factor	(57)
**17q24.3**	rs11655237	LINC00673	C>T	Risk factor	(58)
**12q15**	rs968697	HMGA2	T>C	Risk factor	(52)
**11p15.5**	rs2839698	H19	G>A	Risk factor	(59)
**11p15.5**	rs3024270	H19	C>G	Risk factor	(59)
**11p15.5**	rs217727	H19	G>A	Protective factor	(59)
**20q13**	rs6090311	YTHDF1	A>G	Protective factor	(60)
**6q21**	rs9404590	LIN28B	T>G	Risk factor	(61)
**6q21**	rs314276	LIN28B	C>A	Risk factor	(61)
**6q25**	rs7766006	WTAP	G>T	Protective factor	(62)
**3p25.3**	rs23795	hOGG1	A>G	Risk factor	

### Cancer-Related Genes

Pakakasama et al. addressed the association between myeloperoxidase (MPO) promotor gene polymorphism located on chromosome 17q23 and hepatoblastoma in 2003 (56). They demonstrated that MPO-463 G>A was associated with the reduced susceptibility of hepatoblastoma. Their study represents the first case-control study regarding genetic polymorphism and hepatoblastoma risk, although the cases were only less than 100. The significant roles of MPO-463 G>A polymorphism were also reported in other cancers, including cervical, lung, breast, and bladder cancer (63–66). MPO is an oxidative enzyme located in neutrophils and monocytes. It can catalyze an oxidation reaction to generate hypochlorous acid (HOCl), which is involved in DNA damage and inhibition of DNA repair (67). Carrying G/A or A/A genotype affects the expression of MPO and reduces the generation of oxygen radicals to decrease the risk of cancer.

In the following year, Pakakasama et al. conducted another study to elaborate that *CCND1* gene rs9344 G>A polymorphism affecting gene splicing was associated with the age of onset of hepatoblastoma (57). *CCND1* was identified as the core gene in the β-catenin/LEF pathway, which is relevant to hepatoblastoma’s development (68, 69). The same as the study mentioned above, the cases of these two studies were less than 100, which limited the reliability of the statistical result in the subgroups. In order to affirm these conclusions, study subjects are supposed to enlarge in a future study.

### *RAS* Gene

More than a decade after that, the progression of studies about SNPs and hepatoblastoma was stagnant. In 2019, the third study, a relatively large-scale case-control study that recruited 213 cases in Chinese children, was conducted by our research group (70). As a famous oncogenic role, the *RAS* gene (*KRAS*, *NRAS*, and *HRAS*) is commonly mutated in human cancers (71–73). However, in this study, we regrettably identified that one *NRAS* polymorphism and three *KRAS* polymorphisms do not correlate with hepatoblastoma susceptibility.

### Long Non-coding RNAs (lncRNAs)

LncRNAs, with over 200 nucleotides in length and involved in diverse gene regulation, account for a large number of ncRNAs (74). Various studies have verified that lncRNAs play a vital role in transcription processes, regulation of cellular contexts, assembly of protein, tumor suppressor dysregulation, and other crucial biological function (75–80). *LINC00673*, located on chromosome 17q24.3, has been reported as an oncogene in diverse cancers (81–83). Childs et al. performed a genome-wide association study (GWAS) to confirm that *LINC00673* rs11655237 polymorphism is associated with pancreatic cancer susceptibility (84). Considering the involvement of *LINC00673* in the occurrence and development of diverse cancers while there were no previous studies linking *LINC00673* to hepatoblastoma, our research group conducted a case-control study selecting this polymorphism and confirmed that the *LINC00673* rs11655237 C>T polymorphism may be correlated with hepatoblastoma susceptibility. In the stratified analysis, significant result was also found in the subgroup of clinical stages III+IV (58). The patients carrying this SNP seemed to tend to suffer severe hepatoblastoma. The conjecture based on the statistical result needs further validation.

LncRNA *H19* gene, a maternally imprinted gene, is located on chromosome 11p5.5 and highly expressed during the stage of embryonic development (85, 86). H19 plays a vital role in tumorigenesis and the development of malignant tumors *via* regulation of transcription (87). Tan et al. identified that rs2839698 G>A and rs3024270 C>G, which decreased long non-coding RNA MRPL23 antisense RNA 1 (MRPL23-AS1) expression, were significantly correlated with increased hepatoblastoma risk. In contrast, rs217727 G>A increased MRPL23-AS1 expression to reduced hepatoblastoma risk in the Han population (59). Carrying GGG and AGG haplotypes (order rs2839698, rs3024270, rs217727), children have a tendency to suffer hepatoblastoma. These three polymorphisms were reported to affect the folding structures of *H19* mRNA (88). These results revealed that even though SNPs are in the same gene, their effects of hepatoblastoma may be different. These SNPs are expected to be the biomarkers of early diagnosis of hepatoblastoma. However, the functions of *H19* polymorphism in hepatoblastoma still need to be further validated.

### LIN28

*LIN28A* and *LIN28B* are two paralogs of *LIN28*, located in chromosome 1p36.11 and 6q21, respectively (89). They can bind to the target RNAs, involved separately or jointly in human development and metabolism, to affect cancer occurrence *via* inhibition of let-7 miRNA (90). Yang et al. enrolled 275 hepatoblastoma cases and 1,018 healthy controls to prove *LIN28B* SNPs (rs94904590 T>G and rs314276 C>A) could increase the risk of hepatoblastoma (61). The *LIN28A* SNP (rs3811464 G>A) in hepatoblastoma affected hepatoblastoma in a low-penetrating manner because the significant result was only found in the stratified analysis (91). Four *LIN28A* SNPs (rs3811464 G>A, rs3811463 T>C, rs34787247 G>A, and rs11247957 G>A) were analyzed in hepatoblastoma, neuroblastoma, and Wilms tumor. Nevertheless, the association between the same SNPs with different malignant tumors is diverse (91–93). Interestingly, these findings suggested that the effects of *LIN28A* polymorphisms were specific in a specific cancer.

### HMGA2

HMGA2, a member of the high mobility group (HMG) proteins family, carries a typical functional sequence motif named AT-hooks (94). HMGA2 regulates gene transcription in a modification of chromatin construction way (95). Moreover, it is mainly expressed in embryonic stem cells during embryogenesis rather than in adult tissue cells (96). Many studies identified that aberrant HMGA2 expression is associated with diverse cancer (94). Li et al. detected that *HMGA2* rs968697 T>C polymorphism was related to hepatoblastoma susceptibility in Chinese children (52).

### Base Excision Repair Pathway Genes

DNA damage is common in humans, and DNA repair systems maintain the stability and integrity of DNA. If the damaged DNA is not repaired, genomic instability may eventually evolve into tumorigenesis (97). The base excision repair (BER) pathway is a critical part of DNA repair systems (98). Zhuo et al. conducted a case-control study exploring the relationship between six BER pathway genes (*PARP1*, *hOGG1*, *FEN1*, *APEX1*, *LIG3*, and *XRCC1*) and hepatoblastoma. *hOGG1* gene rs293795 A>G was significantly correlated with hepatoblastoma risk (99).

### Other Genes

As a tumor suppressor gene studied widely, *TP53* is located on human chromosome 17p13.1 and plays a vital role in apoptosis and tumorigenesis (100). Among genes, *TP53* has the highest pertinence with human tumors. Aberrant expression and dysfunction of *TP53* have been detected in various human tumor cases. The *TP53* rs1042522 C>G polymorphism leads to an amino acid alteration (Arg to Pro) and therefore influences the susceptibility of various malignant tumors (101–103). Our research group conducted two studies to explore the association between *TP53* rs1042522 C>G polymorphism and hepatoblastoma. The significant result could not be found in the first study, which enrolled 213 hepatoblastoma cases and 958 cancer-free controls (104). After enlarging the study subjects to 313 cases and 1,446 controls and adding the analysis of rs4938723 T>C of *miR-34b/c*, Liu et al. did not observe any significant result either (105). *CMYC* is also a critical oncogene and reported that the expression of c-Myc increased in hepatoblastoma tissue (106). However, Yang et al. conducted a study in Chinese children and the result showed that CMYC rs4645943 and rs2070583 polymorphisms were not correlated with hepatoblastoma risk (107).

### N6-Methyladenosine (m^6^A) Modification Genes

Mainly occurring on the N6-position of adenosine, m^6^A as an invertible epigenetic modification is prevalent in various eucaryotes (108). m^6^A prefers appearing in 3’untranslated regions (3’UTRs), around termination codons, and within long internal exons (109). Although m^6^A modification does not disturb base paring or coding, it was reported that it is involved in various RNA metabolism, including RNA expression, alternative splicing, and export, and therefore plays a critical role in tumor occurrence and development (110). Cui et al. demonstrated that the majority of m^6^A-related genes were overexpressed in hepatoblastoma tissues (111). However, at present, the research about m^6^A-related gene polymorphism in hepatoblastoma is few.

m^6^A proteins can be divided into three categories, namely, “writers”, “erasers”, and “readers”, which have the function of adding, removing, and recognizing, respectively (112). Methyltransferase-like 3 (METTL3) is a catalytic enzyme that, combined with methyltransferase-like 14 (METTL14), becomes a heterocomplex; and Wilms’ tumor 1–associated protein (WTAP) as an assistant protein interacts with this heterocomplex (113). *METTL3* was localized at nuclear speckles and was involved in mRNA splicing to regulate mRNA metabolism (110). Liu et al. demonstrated that high expression of *METTL3* is the main factor of the aberration of m^6^A and thereby promotes hepatoblastoma growth *via* the Wnt/β-catenin pathway (114). Downregulation of *METTL14* was demonstrated to correlate with the prognosis of hepatocellular carcinoma (47). In contrast, the expression of *WTAP* was significantly upregulated in hepatocellular carcinoma (115). m^6^A demethylases, known as “erasers”, include obesity-associated protein (FTO) and alkylation repair homolog protein 5 (ALKBH5). FTO and ALKBH5 are α-ketoglutarate (α-KG) and Fe (II)-dependent demethylases that remove the RNA m^6^A modification (116). There are many kinds of “readers” enzymes including YTH domain-containing family (YTHDF1-3 and YTHDC1-2), HNRNPA2BFHU1, Mrb1, ELAVL1, IGF2BPs, and eIFs. “Writers”, as binding proteins with the function of specific recognition, impact the gene expression after RNA transcription (117).

**Figure 1:**
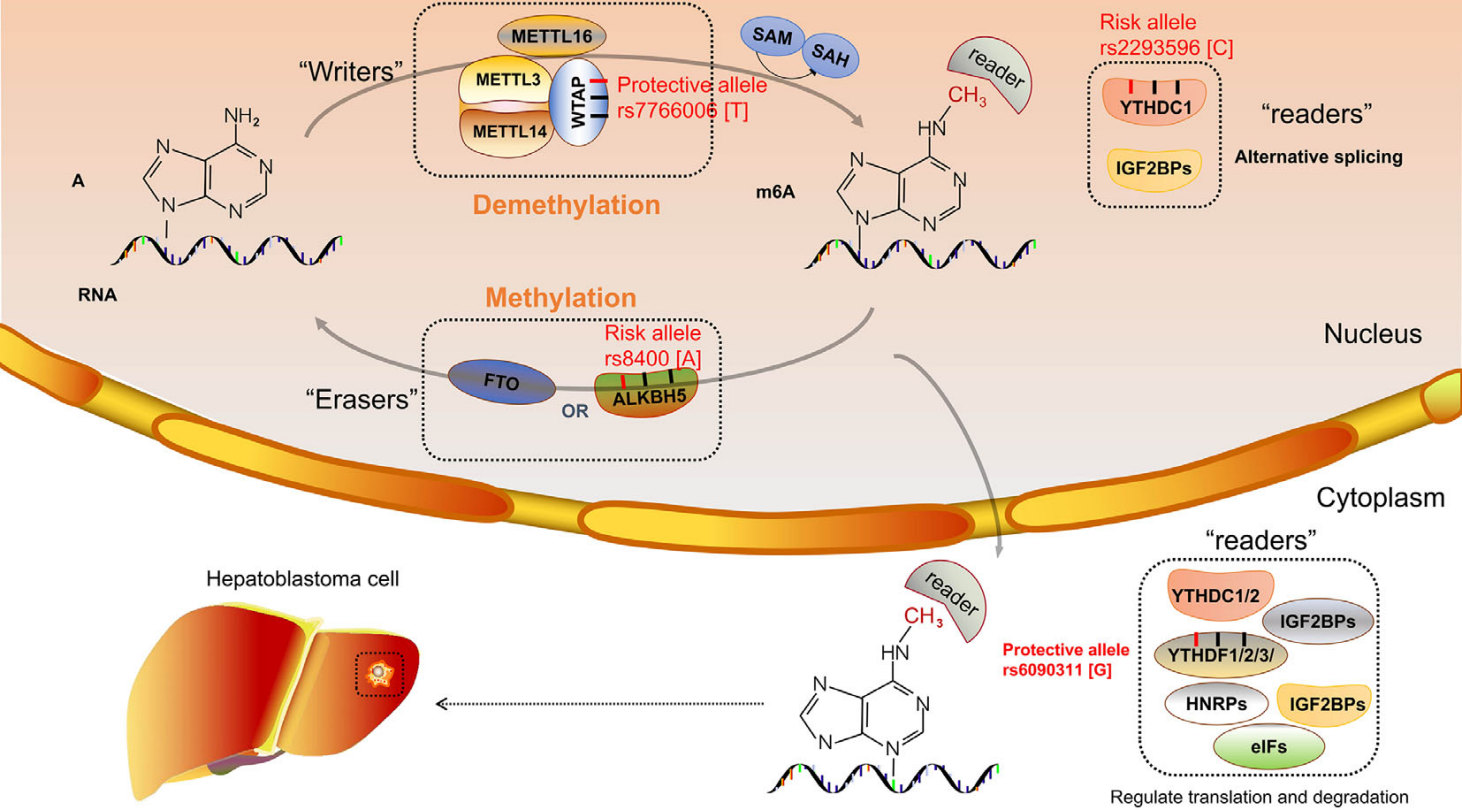
N6-methyladenosine (m^6^A) modification core gene polymorphisms and hepatoblastoma risk.

To our knowledge, only our research group investigated the relationship between m^6^A modification core gene polymorphisms and hepatoblastoma risk. Several research results have been published. Enrolled 313 cases and 1,446 controls, a Chinese seven-center case-control study was conducted. The *WTAP* rs7766006 G>T was significantly correlated with reduced hepatoblastoma risk. Preliminary annotation revealed that *WTAP* mRNA levels were upregulated in the liver in those who carried the rs7766006 T genotype (62). The *YTHDF1* rs6090311 G allele was identified as a protective factor of hepatoblastoma, and expression quantitative trait loci (eQTL) analyses showed that rs6090311 A>G might affect the mRNA level (60). Similar studies detected the association between *YTHDC1* and *ALKBH5* polymorphism and hepatoblastoma. However, significant relationships between *YTHDC1* rs2293596 T>C and *ALKBH5* rs8400 G>A polymorphism and hepatoblastoma risk were observed in the subgroup of clinical stage III+IV in stratification analysis (118, 119), requiring further study to identify whether these SNPs correlate to the prognosis of hepatoblastoma. Results indicated that the m^6^A gene SNPs might affect the m^6^A modification and thereby influence the hepatoblastoma growth (**Figure 1**). Our research group has also conducted the study of *METTL3* and *METTL14* polymorphisms and hepatoblastoma risk, and these results await to be published.

Considering the importance of m^6^A modification in malignant tumors, the studies of m^6^A modification core gene polymorphisms and hepatoblastoma risk are still inadequate. Besides, the number of SNPs included in the research is not enough, and more SNPs are urging to be enrolled to analyze.

## Discussion and Future Directions

The treatment approaches of hepatoblastoma mainly include chemotherapy, surgical resection, and liver transplantation. In order to avoid overtreatments and improve the efficiency of treatment, individualized approaches are needed to provide to patients with different conditions. According to the recommendation of International Childhood Liver Tumor Strategy Group (SIOPEL), patients with high risk are suggested to be given dose-dense cisplatin weekly (120). Up to now, only alpha-fetoprotein (AFP) is used as a biomarker in clinics (121). If more biomarkers are applied in clinical practice for early diagnosis, the survival rate could be vastly improved. We reviewed the development of molecular epidemiology of hepatoblastoma. Advances in genotyping technologies facilitate the measuring of polymorphisms in a mass of samples. The sample sizes are finite in previous studies. However, the sample sizes have been distinctly enlarged with the launching of multicenter studies.

There some limitations of the research that actually need to be addressed. In recent years, the study subjects mentioned above are all from the Han population. Therefore, the results could not be generalized to other ethnicities. The precise functional role of SNPs in hepatoblastoma still awaits to be explored. Besides, there are plenty of cancer-related gene polymorphisms waiting to be detected.

In the past decades, genome-wide association study (GWAS), which is a method of conducting high-throughput sequencing technologies to measure plenty of polymorphisms, has discovered abundant significant cancer-related loci to improve the methods of genetic research (122, 123). This technology was applied in other childhood solid tumors such as neuroblastoma and has determined multiple disease-related loci (124, 125). GWAS needs a larger sample to apply in multistages and multicenters to ensure the reliability of the results. Based on the result that abundant SNPs were detected by GWAS, it is challenging to seek out the actual cancer-related loci and explain their biological function in hepatoblastoma. Identifying SNPs with important functionalities could be applied in prenatal screening to diminish birth defects. There is no doubt that the application of GWAS in hepatoblastoma is necessary. It will contribute to figuring out the biomarkers of hepatoblastoma and guiding people to understand hepatoblastoma’s etiology to improve the prevention and treatment of hepatoblastoma.

## Author Contributions

LM and ZZ: conceptualization and supervision. HC and QG: resources. HC: writing—original draft preparation. QG, HG, and LM: writing—review and editing. ZZ: project administration. LM and ZZ: funding acquisition. All authors contributed to the article and approved the submitted version.

## Funding

This study was supported by grants from the National Natural Science Foundation of China (nos. 82002636, 82002635) and China Postdoctoral Science Foundation (nos. 2020T130132, 2020M682668).

## Conflict of Interest

The authors declare that the research was conducted in the absence of any commercial or financial relationships that could be construed as a potential conflict of interest.
